# Crystal structure of (*S*)-2-[(3*S*,8*S*,9*S*,10*R*,13*S*,14*S*,17*R*)-3-hy­droxy-10,13-dimethyl-2,3,4,7,8,9,10,11,12,13,14,15,16,17-tetra­deca­hydro-1*H*-cyclo­penta[*a*]phenanthren-17-yl]-*N*-meth­oxy-*N*-methyl­pro­pan­amide (Fernholz Weinreb amide)

**DOI:** 10.1107/S2056989015001747

**Published:** 2015-02-18

**Authors:** Elvar Ørn Viktorsson, Ove Alexander Høgmoen Åstrand, Rasha Sabah Haseeb, Carl Henrik Görbitz, Pål Rongved

**Affiliations:** a School of Pharmacy, University of Oslo, PO Box 1068 Blindern, N-0316 Oslo, Norway; b Department of Chemistry, University of Oslo, PO Box 1033 Blindern, N-0315 Oslo, Norway

**Keywords:** crystal structure, Liver *X* receptor, obesity, steroid

## Abstract

The literature compound 3β-hy­droxy-bis­nor-5-cholenic aldehyde is an important inter­mediate for the synthesis of new modulators of the nuclear oxysterol receptor Liver *X*. As part of our ongoing search for new LXR antagonists, the title compound, C_24_H_39_NO_3_, has proven to be an important inter­mediate in our new synthetic pathway, giving the corresponding aldehyde in high yield and in only three steps from the commercially available 3β-hy­droxy-bis­nor-5-cholenic acid. The title amide crystallized with two mol­ecules in the asymmetric unit, linked into helices by O—H⋯O hydrogen bonds involving the hy­droxy and carbonyl groups.

## Abstract 2

In research towards new antagonists against the Liver *X* receptor, the important inter­mediate Fernholz acid Weinreb amide has been synthesized and characterized.

## Chemical context   

In the nuclear receptor (NR) family, the two isoforms of the nuclear oxysterol receptor Liver *X* (LXRα and LXRβ) are emerging new drug targets. They are key players for a number of important processes related to disease, such as metabolic and cardiovascular diseases, lipid metabolism, inflammation and cancer (Steffensen & Gustafsson, 2006 ▸; Laffitte *et al.*, 2003 ▸). LXR modulators have been investigated as potential drugs in the therapy of cardiovascular diseases, metabolic syndrome, regulation of inflammatory response and immunity, skin diseases and are effective in the treatment of murine models of atherosclerosis, diabetes and Alzheimer’s disease (Viennois *et al.*, 2011 ▸, 2012 ▸; Jakobsson *et al.*, 2012 ▸). Further, such agents have been shown to affect anti-inflammatory activity (Zhu & Bakovic, 2008 ▸; Zhu *et al.*, 2012 ▸; Solan *et al.*, 2011 ▸) and cell proliferation in a number of major cancer forms such as LNCaP human prostate cancer cells. (Viennois *et al.*, 2012 ▸; Jakobsson *et al.*, 2012 ▸). The ligand-binding pocket (LBP) of LXR allows binding of side-chain-oxygenated sterols (OHCs).

Recently, OHCs with a specific stereochemistry at the 23-hy­droxy­ated side-chain carbon have also been shown to regulate the Hedgehog signalling pathway (Hh), a key developmental pathway playing multiple roles in embryonic development, including stem-cell differentiation (Corman *et al.*, 2012 ▸). In our drug-design programme, our retrosynthetic analysis for the establishment of synthetic routes to the pharmacophores in different OHCs revealed that the aldehyde analogue of the title compound [Fernholz aldehyde, (II)] is a key compound leading to a number of new library candidates for biological testing (Åstrand *et al.*, 2014*a* ▸,*b* ▸). We have now identified the title compound, Fernholz Weinreb amide (I), as a new key inter­mediate to the Fernholz aldehyde, reducing the number of steps in the stereoselective synthesis. The O-TBDMS-protected Weinreb amide (I) may be used to prepare (II) using DIBALH, transferred to ketones with Grignard reagents or used for other synthetic transformations (Sivaraman *et al.*, 2009 ▸; Davies *et al.*, 2013 ▸). 
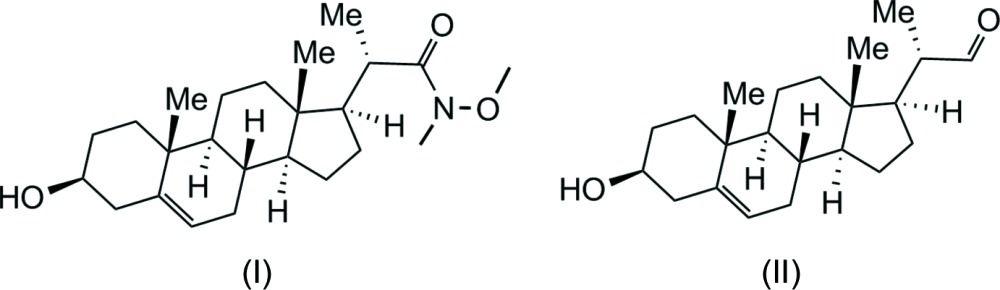


## Structural commentary   

**Figure 1:**
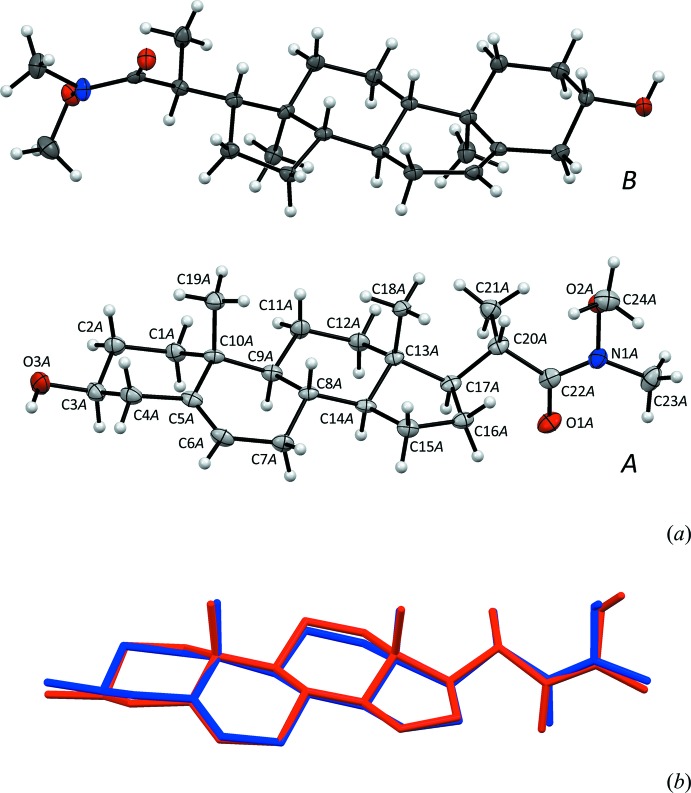
(*a*) The asymmetric unit of (I), showing the two mol­ecules *A* (light grey C atoms and atomic labels included) and *B* (dark C atoms). (*b*) An overlay between mol­ecules *A* (blue) and *B* (red), with an r.m.s. value of 0.300 Å. H atoms have been omitted in (b).

The asymmetric unit of (I), with two independent mol­ecules *A* and *B*, is depicted in Fig. 1 ▸ *a*. The macrocyclic part of (I) is also found in the naturally occurring hormone cholesterol and in close to 250 other steroids in the Cambridge Structural Database (CSD; Version 5.35 of November 2013; Groom & Allen, 2014 ▸). The mol­ecular conformation of this part of the mol­ecule is rigid, as shown from the overlay between *A* and *B* in Fig. 1 ▸ *b*. If the substituent at C17 is not included, the fit improves from 0.300 to 0.173 Å. Compound (I) also shares the hy­droxy group at C3 with cholesterol, but the *N*-meth­oxy-*N*-methyl­propanamide functionality has not previously been introduced into steroids; only the structure of the parent carb­oxy­lic acid has been reported previously (CSD refcode HAHSAL; Kurek-Tyrlik *et al.*, 2004 ▸).

## Supra­molecular features   

**Figure 2:**
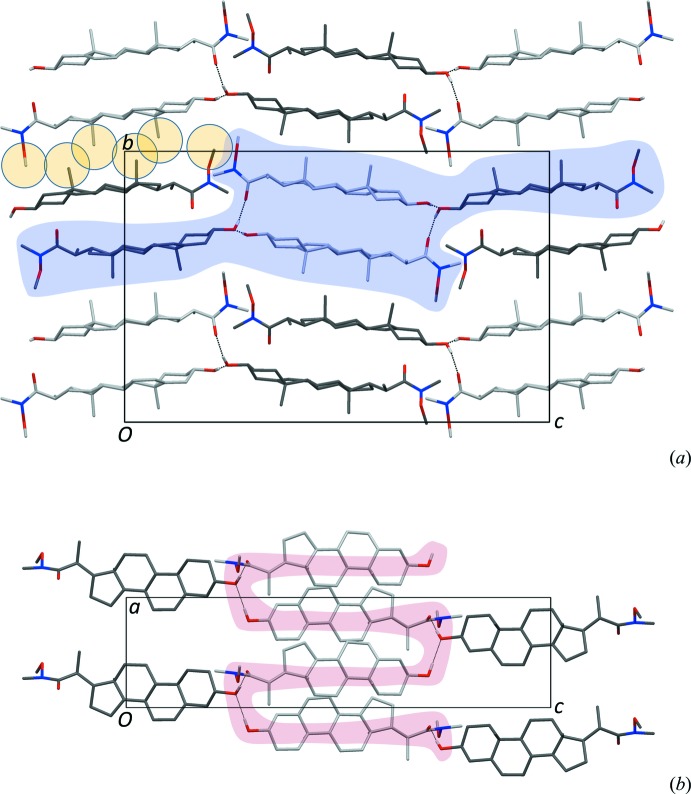
(*a*) Unit-cell and crystal packing viewed along the *a* axis. The colour coding is as in Fig. 1 ▸. The orange circles highlight a series of methyl groups; the blue area shades a hydrogen-bonded chain in shape of a flat helix. The chain, as a pink shape, is shown in more detail in (*b*) (the view is along the *b* axis).

The unit-cell and the mol­ecular packing of (I) are shown in Fig. 2 ▸. As a class, steroids display a pronounced tendency to form crystal structures with more than one mol­ecule in the asymmetric unit; *e.g.* for about 35% of the 250 compounds mentioned above. The maximum *Z*′ value of 16 is reached for the high-temperature polymorph of cholesterol itself (CHOEST21: Hsu *et al.*, 2002 ▸). Compound (I) has a *Z*′ value of 2, the two mol­ecules differing in the way the hy­droxy groups make inter­molecular hydrogen bonds (Table 1 ▸). Only the carbonyl group of mol­ecule *A* is an acceptor, while the hy­droxy groups of the *B* mol­ecules are both donors and acceptors and thus serve to link adjacent *A* mol­ecules along the *a* axis. In this process, stacks of either *A* or *B* mol­ecules along the *a* axis expose all the methyl groups on the outside, giving distinct regions with meth­yl–methyl inter­actions (Fig. 2 ▸ *a*). This is not a common mol­ecular aggregation pattern for steroids, but some related *Z*′ = 2 structures were found in the CSD, all hydrates without additional hydrogen-bond donors or acceptors in their C17 substituents (KESNAX: Sheng-Zhi *et al.*, 1990 ▸; ZZZNVG01: Jiang *et al.*, 2001 ▸; XOSLOH: Subash-Babu *et al.*, 2009 ▸).

## Synthesis and crystallization   

Compound (I) (348 mg) was dissolved in a minimum amount of boiling EtOAc (40 ml). The flask containing the solution was wrapped in aluminium foil and left overnight at room temperature to afford colourless crystalline needles.

## Refinement   

Crystal data, data collection and structure refinement details are summarized in Table 2 ▸. Coordinates were refined for hydroxyic H atoms; other H atoms were positioned with idealized geometry with fixed C—H = 0.95 (aromatic), 0.98 (meth­yl), 0.99 (methyl­ene) or 1.00 Å (methine) Å. *U*_iso_ values were set to 1.2*U*_eq_ of the carrier atom, or 1.5*U*_eq_ for methyl and hy­droxy groups.

## Supplementary Material

Crystal structure: contains datablock(s) I, global. DOI: 10.1107/S2056989015001747/hb7343sup1.cif

Structure factors: contains datablock(s) I. DOI: 10.1107/S2056989015001747/hb7343Isup2.hkl

Supporting information file. DOI: 10.1107/S2056989015001747/hb7343Isup3.cml

CCDC reference: 1045692

Additional supporting information:  crystallographic information; 3D view; checkCIF report

## Figures and Tables

**Table 1:** Hydrogen-bond geometry (, )

*D*H*A*	*D*H	H*A*	*D* *A*	*D*H*A*
O3*A*H3*A*O3*B* ^i^	0.86(4)	1.93(4)	2.782(4)	180(5)
O3*B*H3*B*O1*A* ^ii^	0.83(4)	1.95(4)	2.768(3)	169(4)

Symmetry codes: (i) 
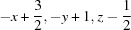
; (ii) 
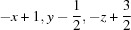
.

**Table 2:** Experimental details

Crystal data	
Chemical formula	C_24_H_39_NO_3_
*M*_r_	389.56
Crystal system, space group	Orthorhombic, *P*2_1_2_1_2_1_
Temperature (K)	105
*a*, *b*, *c* ()	7.7256(4), 19.0030(9), 29.8162(15)
*V* (^3^)	4377.3(4)
*Z*	8
Radiation type	Mo *K*
(mm^1^)	0.08
Crystal size (mm)	0.65 0.21 0.10

Data collection	
Diffractometer	Bruker D8 Vantage single-crystal CCD
Absorption correction	Multi-scan (*SADABS*; Bruker, 2013 ▸)
*T*_min_, *T*_max_	0.852, 1.000
No. of measured, independent and observed [*I* > 2(*I*)] reflections	44800, 7739, 5760
*R*_int_	0.089
(sin /)_max_ (^1^)	0.596

Refinement	
*R*[*F* ^2^ > 2(*F* ^2^)], *wR*(*F* ^2^), *S*	0.048, 0.098, 1.04
No. of reflections	7739
No. of parameters	511
H-atom treatment	H atoms treated by a mixture of independent and constrained refinement
_max_, _min_ (e ^3^)	0.21, 0.19

Computer programs: *APEX2* and *SAINT-Plus* (Bruker, 2013 ▸), *SHELXS2013* (Sheldrick, 2008 ▸), *SHELXL2013* (Sheldrick, 2015 ▸) and *Mercury* (Macrae *et al.*, 2008 ▸).

## supplementary crystallographic information

### Crystal data

**Table:**

C_24_H_39_NO_3_	*D*_x_ = 1.182 Mg m^−^^3^
*M**_r_* = 389.56	Mo *K*α radiation, λ = 0.71073 Å
Orthorhombic, *P*2_1_2_1_2_1_	Cell parameters from 9981 reflections
*a* = 7.7256 (4) Å	θ = 2.3–24.9°
*b* = 19.0030 (9) Å	µ = 0.08 mm^−^^1^
*c* = 29.8162 (15) Å	*T* = 105 K
*V* = 4377.3 (4) Å^3^	Flat needle, colourless
*Z* = 8	0.65 × 0.21 × 0.10 mm
*F*(000) = 1712	

### Data collection

**Table:**

Bruker D8 Vantage single-crystal CCD diffractometer	7739 independent reflections
Radiation source: fine-focus sealed tube	5760 reflections with *I* > 2σ(*I*)
Graphite monochromator	*R*_int_ = 0.089
Detector resolution: 8.3 pixels mm^-1^	θ_max_ = 25.1°, θ_min_ = 2.3°
Sets of exposures each taken over 0.5° ω rotation scans	*h* = −9→9
Absorption correction: multi-scan (*SADABS*; Bruker, 2013)	*k* = −22→22
*T*_min_ = 0.852, *T*_max_ = 1.000	*l* = −35→35
44800 measured reflections	

### Refinement

**Table:**

Refinement on *F*^2^	0 restraints
Least-squares matrix: full	Hydrogen site location: inferred from neighbouring sites
*R*[*F*^2^ > 2σ(*F*^2^)] = 0.048	H atoms treated by a mixture of independent and constrained refinement
*wR*(*F*^2^) = 0.098	*w* = 1/[σ^2^(*F*_o_^2^) + (0.0448*P*)^2^ + 0.1501*P*] where *P* = (*F*_o_^2^ + 2*F*_c_^2^)/3
*S* = 1.04	(Δ/σ)_max_ < 0.001
7739 reflections	Δρ_max_ = 0.21 e Å^−^^3^
511 parameters	Δρ_min_ = −0.19 e Å^−^^3^

### Special details

**Table:**

Geometry. All e.s.d.'s (except the e.s.d. in the dihedral angle between two l.s. planes) are estimated using the full covariance matrix. The cell e.s.d.'s are taken into account individually in the estimation of e.s.d.'s in distances, angles and torsion angles; correlations between e.s.d.'s in cell parameters are only used when they are defined by crystal symmetry. An approximate (isotropic) treatment of cell e.s.d.'s is used for estimating e.s.d.'s involving l.s. planes.
Refinement. No constraints or restraints applied

### Fractional atomic coordinates and isotropic or equivalent isotropic displacement parameters (Å^2^)

**Table:**

	*x*	*y*	*z*	*U*_iso_*/*U*_eq_	
O1A	0.8266 (3)	0.67664 (11)	0.71479 (7)	0.0314 (6)	
O2A	0.7377 (3)	0.50014 (11)	0.73658 (6)	0.0268 (6)	
O3A	0.7896 (3)	0.69062 (13)	0.28850 (7)	0.0321 (6)	
H3A	0.893 (6)	0.697 (2)	0.2790 (13)	0.048*	
N1A	0.8127 (4)	0.56722 (14)	0.74205 (8)	0.0262 (7)	
C1A	0.6249 (5)	0.67785 (17)	0.40586 (10)	0.0234 (8)	
H1A	0.5099	0.6633	0.4171	0.028*	
H2A	0.6438	0.7272	0.4153	0.028*	
C2A	0.6223 (5)	0.67502 (18)	0.35440 (10)	0.0254 (8)	
H4A	0.5928	0.6268	0.3444	0.030*	
H5A	0.5327	0.7075	0.3428	0.030*	
C3A	0.7964 (5)	0.69551 (17)	0.33605 (10)	0.0250 (8)	
H31A	0.8210	0.7454	0.3446	0.030*	
C4A	0.9375 (5)	0.64851 (17)	0.35544 (10)	0.0256 (8)	
H41A	0.9215	0.6001	0.3440	0.031*	
H42A	1.0515	0.6655	0.3449	0.031*	
C5A	0.9374 (5)	0.64668 (16)	0.40622 (10)	0.0202 (8)	
C6A	1.0815 (5)	0.65764 (16)	0.42912 (11)	0.0243 (8)	
H61A	1.1832	0.6685	0.4126	0.029*	
C7A	1.0964 (4)	0.65414 (17)	0.47918 (10)	0.0233 (8)	
H71A	1.1122	0.7023	0.4911	0.028*	
H72A	1.2005	0.6264	0.4872	0.028*	
C8A	0.9385 (4)	0.62097 (16)	0.50128 (10)	0.0172 (7)	
H81A	0.9438	0.5689	0.4968	0.021*	
C9A	0.7721 (4)	0.64902 (16)	0.47901 (10)	0.0195 (7)	
H91A	0.7785	0.7015	0.4811	0.023*	
C10A	0.7637 (4)	0.63144 (15)	0.42821 (10)	0.0190 (7)	
C11A	0.6082 (4)	0.62733 (18)	0.50481 (10)	0.0242 (8)	
H11A	0.5089	0.6543	0.4928	0.029*	
H11C	0.5855	0.5769	0.4990	0.029*	
C12A	0.6168 (4)	0.63881 (17)	0.55576 (10)	0.0230 (8)	
H12A	0.6176	0.6899	0.5621	0.028*	
H12C	0.5122	0.6184	0.5699	0.028*	
C13A	0.7778 (4)	0.60512 (15)	0.57639 (10)	0.0183 (7)	
C14A	0.9350 (4)	0.63605 (16)	0.55146 (10)	0.0181 (8)	
H14A	0.9255	0.6883	0.5545	0.022*	
C15A	1.0903 (4)	0.61434 (16)	0.57969 (10)	0.0224 (8)	
H15A	1.1881	0.6473	0.5754	0.027*	
H15C	1.1289	0.5662	0.5719	0.027*	
C16A	1.0214 (4)	0.61751 (17)	0.62834 (11)	0.0229 (8)	
H16A	1.0735	0.6578	0.6445	0.027*	
H16C	1.0508	0.5737	0.6446	0.027*	
C17A	0.8214 (4)	0.62640 (16)	0.62533 (10)	0.0202 (8)	
H17A	0.7948	0.6776	0.6287	0.024*	
C18A	0.7693 (5)	0.52436 (15)	0.57226 (10)	0.0219 (8)	
H18A	0.7630	0.5112	0.5405	0.033*	
H18C	0.6664	0.5069	0.5879	0.033*	
H18D	0.8732	0.5036	0.5857	0.033*	
C19A	0.7198 (5)	0.55331 (16)	0.42009 (10)	0.0277 (9)	
H19A	0.7365	0.5420	0.3883	0.042*	
H19C	0.5991	0.5445	0.4284	0.042*	
H19D	0.7961	0.5237	0.4384	0.042*	
C20A	0.7291 (4)	0.58734 (16)	0.66317 (10)	0.0223 (8)	
H20A	0.7572	0.5361	0.6608	0.027*	
C21A	0.5318 (4)	0.59589 (18)	0.66345 (11)	0.0285 (9)	
H21A	0.4833	0.5750	0.6362	0.043*	
H21C	0.5025	0.6460	0.6645	0.043*	
H21D	0.4836	0.5721	0.6898	0.043*	
C22A	0.7925 (4)	0.61417 (17)	0.70820 (10)	0.0234 (8)	
C23A	0.8338 (5)	0.58740 (19)	0.78883 (11)	0.0340 (9)	
H23A	0.9119	0.5541	0.8037	0.051*	
H23C	0.7209	0.5868	0.8038	0.051*	
H23D	0.8829	0.6349	0.7905	0.051*	
C24A	0.8716 (5)	0.44840 (17)	0.73243 (12)	0.0329 (9)	
H24A	0.8205	0.4012	0.7337	0.049*	
H24C	0.9545	0.4540	0.7570	0.049*	
H24D	0.9314	0.4546	0.7037	0.049*	
O1B	0.3248 (3)	0.29218 (11)	0.34061 (7)	0.0241 (6)	
O2B	0.1000 (3)	0.43877 (11)	0.30237 (7)	0.0244 (6)	
O3B	0.3740 (3)	0.28938 (12)	0.75771 (7)	0.0275 (6)	
H3B	0.308 (5)	0.2590 (19)	0.7683 (11)	0.041*	
N1B	0.2268 (4)	0.38484 (12)	0.30187 (8)	0.0212 (6)	
C1B	0.1604 (4)	0.30886 (16)	0.64465 (9)	0.0203 (8)	
H1B	0.0411	0.3224	0.6362	0.024*	
H2B	0.1808	0.2606	0.6333	0.024*	
C2B	0.1737 (4)	0.30804 (17)	0.69563 (10)	0.0222 (8)	
H4B	0.1448	0.3552	0.7076	0.027*	
H5B	0.0897	0.2739	0.7080	0.027*	
C3B	0.3537 (4)	0.28808 (16)	0.70983 (10)	0.0204 (8)	
H31B	0.3798	0.2396	0.6987	0.024*	
C4B	0.4852 (4)	0.33881 (16)	0.69055 (10)	0.0216 (8)	
H41B	0.6031	0.3220	0.6980	0.026*	
H42B	0.4695	0.3855	0.7047	0.026*	
C5B	0.4690 (4)	0.34639 (16)	0.63992 (10)	0.0167 (8)	
C6B	0.6090 (4)	0.34157 (15)	0.61433 (10)	0.0190 (8)	
H61B	0.7165	0.3331	0.6288	0.023*	
C7B	0.6103 (4)	0.34850 (16)	0.56420 (10)	0.0181 (7)	
H71B	0.6346	0.3018	0.5508	0.022*	
H72B	0.7053	0.3806	0.5554	0.022*	
C8B	0.4407 (4)	0.37637 (15)	0.54505 (10)	0.0148 (7)	
H81B	0.4377	0.4287	0.5487	0.018*	
C9B	0.2861 (4)	0.34433 (15)	0.56994 (9)	0.0148 (7)	
H91B	0.2973	0.2922	0.5666	0.018*	
C10B	0.2883 (4)	0.35925 (15)	0.62135 (9)	0.0164 (7)	
C11B	0.1142 (4)	0.36437 (16)	0.54751 (9)	0.0186 (8)	
H11B	0.0926	0.4151	0.5525	0.022*	
H11E	0.0194	0.3379	0.5622	0.022*	
C12B	0.1090 (4)	0.34958 (17)	0.49675 (10)	0.0195 (8)	
H12B	0.1125	0.2981	0.4917	0.023*	
H12E	−0.0013	0.3675	0.4843	0.023*	
C13B	0.2603 (4)	0.38405 (15)	0.47190 (10)	0.0159 (7)	
C14B	0.4271 (4)	0.35862 (16)	0.49529 (10)	0.0159 (7)	
H14B	0.4238	0.3061	0.4937	0.019*	
C15B	0.5736 (4)	0.38110 (16)	0.46425 (10)	0.0185 (8)	
H15B	0.6739	0.3489	0.4670	0.022*	
H15E	0.6119	0.4296	0.4711	0.022*	
C16B	0.4928 (4)	0.37679 (17)	0.41675 (10)	0.0190 (8)	
H16B	0.5038	0.4225	0.4011	0.023*	
H16E	0.5516	0.3402	0.3987	0.023*	
C17B	0.2998 (4)	0.35775 (15)	0.42354 (9)	0.0163 (7)	
H17B	0.2924	0.3052	0.4242	0.020*	
C18B	0.2422 (5)	0.46448 (14)	0.47231 (10)	0.0205 (8)	
H18B	0.2399	0.4813	0.5034	0.031*	
H18E	0.1344	0.4779	0.4572	0.031*	
H18F	0.3407	0.4856	0.4566	0.031*	
C19B	0.2362 (5)	0.43602 (15)	0.63154 (10)	0.0238 (8)	
H19B	0.1118	0.4420	0.6264	0.036*	
H19E	0.3007	0.4679	0.6118	0.036*	
H19F	0.2632	0.4470	0.6629	0.036*	
C20B	0.1861 (4)	0.38264 (15)	0.38456 (10)	0.0171 (7)	
H20B	0.1963	0.4349	0.3819	0.021*	
C21B	−0.0056 (4)	0.36319 (18)	0.38993 (10)	0.0241 (8)	
H21B	−0.0159	0.3127	0.3961	0.036*	
H21E	−0.0680	0.3745	0.3622	0.036*	
H21F	−0.0554	0.3899	0.4149	0.036*	
C22B	0.2502 (4)	0.34904 (16)	0.34128 (10)	0.0180 (7)	
C23B	0.2346 (5)	0.34870 (17)	0.25866 (10)	0.0297 (9)	
H23B	0.3161	0.3093	0.2606	0.045*	
H23E	0.2735	0.3817	0.2355	0.045*	
H23F	0.1194	0.3309	0.2509	0.045*	
C24B	0.1771 (5)	0.50416 (17)	0.28946 (11)	0.0323 (9)	
H24B	0.2626	0.5182	0.3120	0.048*	
H24E	0.0872	0.5404	0.2872	0.048*	
H24F	0.2342	0.4987	0.2603	0.048*	

### Atomic displacement parameters (Å^2^)

**Table:**

	*U*^11^	*U*^22^	*U*^33^	*U*^12^	*U*^13^	*U*^23^
O1A	0.0366 (16)	0.0248 (14)	0.0329 (14)	0.0014 (12)	−0.0019 (12)	−0.0128 (10)
O2A	0.0243 (14)	0.0266 (13)	0.0295 (13)	−0.0025 (12)	0.0005 (11)	−0.0031 (10)
O3A	0.0298 (16)	0.0442 (15)	0.0224 (14)	−0.0058 (13)	0.0051 (12)	0.0016 (11)
N1A	0.0296 (19)	0.0282 (16)	0.0207 (16)	−0.0023 (14)	0.0012 (14)	−0.0059 (13)
C1A	0.018 (2)	0.027 (2)	0.025 (2)	0.0001 (16)	0.0036 (16)	0.0005 (14)
C2A	0.021 (2)	0.028 (2)	0.027 (2)	−0.0012 (17)	0.0015 (16)	0.0008 (15)
C3A	0.030 (2)	0.0219 (18)	0.0228 (19)	−0.0021 (16)	0.0047 (17)	−0.0006 (14)
C4A	0.024 (2)	0.0242 (19)	0.028 (2)	−0.0035 (17)	0.0075 (16)	−0.0017 (15)
C5A	0.020 (2)	0.0133 (17)	0.028 (2)	0.0011 (16)	0.0059 (17)	−0.0014 (14)
C6A	0.016 (2)	0.0227 (19)	0.034 (2)	0.0007 (16)	0.0084 (18)	0.0027 (15)
C7A	0.0145 (19)	0.0252 (18)	0.030 (2)	−0.0010 (16)	0.0016 (16)	0.0002 (15)
C8A	0.0149 (18)	0.0132 (16)	0.0235 (19)	0.0002 (15)	0.0008 (15)	−0.0028 (13)
C9A	0.0172 (19)	0.0161 (16)	0.0253 (18)	0.0005 (15)	0.0024 (16)	−0.0020 (14)
C10A	0.0140 (18)	0.0156 (16)	0.0274 (18)	−0.0007 (15)	0.0015 (16)	−0.0008 (13)
C11A	0.016 (2)	0.033 (2)	0.024 (2)	−0.0011 (17)	−0.0005 (16)	0.0009 (15)
C12A	0.015 (2)	0.0266 (19)	0.027 (2)	0.0000 (16)	0.0030 (16)	−0.0003 (15)
C13A	0.0190 (19)	0.0149 (16)	0.0210 (18)	0.0003 (15)	0.0005 (16)	−0.0040 (13)
C14A	0.0151 (19)	0.0112 (17)	0.0279 (19)	−0.0008 (15)	0.0012 (16)	−0.0009 (14)
C15A	0.019 (2)	0.0187 (18)	0.030 (2)	0.0024 (16)	0.0004 (16)	0.0000 (14)
C16A	0.021 (2)	0.0176 (18)	0.030 (2)	0.0004 (15)	−0.0036 (16)	−0.0021 (14)
C17A	0.022 (2)	0.0126 (16)	0.0259 (19)	0.0026 (15)	−0.0009 (16)	−0.0042 (13)
C18A	0.024 (2)	0.0205 (17)	0.0214 (18)	−0.0060 (16)	0.0004 (16)	−0.0035 (13)
C19A	0.033 (2)	0.0234 (18)	0.0266 (19)	−0.0068 (17)	0.0025 (18)	−0.0024 (14)
C20A	0.023 (2)	0.0185 (17)	0.0254 (19)	0.0032 (16)	0.0014 (17)	−0.0062 (14)
C21A	0.026 (2)	0.031 (2)	0.028 (2)	−0.0026 (17)	0.0015 (17)	−0.0005 (16)
C22A	0.018 (2)	0.026 (2)	0.026 (2)	0.0031 (16)	0.0052 (16)	−0.0074 (15)
C23A	0.028 (2)	0.050 (2)	0.024 (2)	0.0018 (19)	0.0016 (18)	−0.0092 (17)
C24A	0.029 (2)	0.028 (2)	0.042 (2)	0.0008 (18)	0.0009 (18)	0.0000 (16)
O1B	0.0315 (15)	0.0202 (12)	0.0206 (12)	0.0058 (11)	−0.0002 (11)	−0.0011 (9)
O2B	0.0215 (14)	0.0249 (12)	0.0269 (13)	0.0064 (11)	0.0017 (11)	0.0063 (10)
O3B	0.0290 (16)	0.0349 (15)	0.0184 (14)	−0.0094 (12)	−0.0034 (11)	0.0058 (10)
N1B	0.0221 (17)	0.0238 (14)	0.0177 (15)	0.0091 (14)	0.0002 (13)	0.0021 (11)
C1B	0.0157 (19)	0.0251 (18)	0.0199 (18)	0.0001 (16)	−0.0014 (15)	0.0018 (14)
C2B	0.021 (2)	0.0228 (18)	0.0226 (19)	−0.0033 (15)	0.0003 (16)	0.0018 (14)
C3B	0.026 (2)	0.0195 (17)	0.0153 (18)	−0.0002 (16)	−0.0022 (15)	0.0001 (14)
C4B	0.021 (2)	0.0216 (19)	0.0224 (19)	−0.0043 (15)	−0.0039 (16)	−0.0014 (14)
C5B	0.019 (2)	0.0121 (17)	0.0191 (18)	−0.0028 (15)	−0.0009 (15)	0.0001 (13)
C6B	0.016 (2)	0.0168 (17)	0.025 (2)	−0.0021 (15)	−0.0064 (16)	0.0027 (14)
C7B	0.0160 (19)	0.0149 (16)	0.0233 (19)	0.0013 (15)	−0.0013 (15)	−0.0015 (13)
C8B	0.0138 (18)	0.0111 (16)	0.0196 (18)	0.0002 (15)	−0.0015 (14)	−0.0002 (13)
C9B	0.0146 (19)	0.0116 (15)	0.0183 (17)	0.0006 (14)	−0.0006 (14)	−0.0002 (12)
C10B	0.0142 (19)	0.0160 (16)	0.0189 (17)	−0.0010 (14)	−0.0013 (15)	−0.0006 (13)
C11B	0.0130 (19)	0.0255 (19)	0.0173 (18)	−0.0004 (15)	0.0012 (15)	−0.0004 (14)
C12B	0.0125 (19)	0.0246 (18)	0.0213 (19)	−0.0004 (16)	−0.0011 (15)	−0.0006 (14)
C13B	0.0136 (18)	0.0156 (16)	0.0184 (17)	0.0006 (15)	−0.0038 (14)	−0.0019 (13)
C14B	0.0148 (18)	0.0118 (16)	0.0210 (18)	−0.0003 (15)	−0.0011 (15)	−0.0009 (13)
C15B	0.0156 (19)	0.0181 (17)	0.0217 (19)	−0.0012 (16)	0.0017 (15)	0.0015 (13)
C16B	0.0165 (19)	0.0193 (18)	0.0214 (18)	−0.0002 (15)	0.0005 (15)	−0.0013 (14)
C17B	0.0161 (19)	0.0133 (16)	0.0195 (17)	0.0013 (14)	−0.0010 (15)	0.0005 (13)
C18B	0.022 (2)	0.0203 (17)	0.0193 (17)	0.0061 (17)	−0.0020 (16)	−0.0027 (13)
C19B	0.028 (2)	0.0229 (17)	0.0202 (18)	0.0055 (18)	0.0004 (16)	−0.0013 (13)
C20B	0.0175 (19)	0.0151 (16)	0.0188 (18)	−0.0015 (14)	0.0012 (15)	0.0011 (13)
C21B	0.019 (2)	0.033 (2)	0.0197 (18)	0.0022 (16)	−0.0010 (15)	0.0005 (15)
C22B	0.0123 (18)	0.0189 (17)	0.0229 (18)	−0.0049 (17)	−0.0040 (15)	−0.0017 (14)
C23B	0.034 (2)	0.036 (2)	0.0190 (18)	0.0055 (19)	−0.0005 (17)	−0.0012 (15)
C24B	0.038 (2)	0.0243 (19)	0.035 (2)	0.0023 (17)	0.0045 (18)	0.0064 (16)

### Geometric parameters (Å, º)

**Table:**

O1A—C22A	1.232 (4)	O1B—C22B	1.225 (4)
O2A—N1A	1.410 (3)	O2B—N1B	1.418 (3)
O2A—C24A	1.433 (4)	O2B—C24B	1.431 (4)
O3A—C3A	1.422 (4)	O3B—C3B	1.436 (4)
O3A—H3A	0.86 (4)	O3B—H3B	0.83 (4)
N1A—C22A	1.356 (4)	N1B—C22B	1.370 (4)
N1A—C23A	1.456 (4)	N1B—C23B	1.461 (4)
C1A—C2A	1.535 (4)	C1B—C2B	1.523 (4)
C1A—C10A	1.540 (4)	C1B—C10B	1.541 (4)
C1A—H1A	0.9900	C1B—H1B	0.9900
C1A—H2A	0.9900	C1B—H2B	0.9900
C2A—C3A	1.503 (5)	C2B—C3B	1.503 (4)
C2A—H4A	0.9900	C2B—H4B	0.9900
C2A—H5A	0.9900	C2B—H5B	0.9900
C3A—C4A	1.523 (5)	C3B—C4B	1.513 (4)
C3A—H31A	1.0000	C3B—H31B	1.0000
C4A—C5A	1.514 (4)	C4B—C5B	1.522 (4)
C4A—H41A	0.9900	C4B—H41B	0.9900
C4A—H42A	0.9900	C4B—H42B	0.9900
C5A—C6A	1.322 (5)	C5B—C6B	1.327 (4)
C5A—C10A	1.521 (5)	C5B—C10B	1.522 (4)
C6A—C7A	1.499 (4)	C6B—C7B	1.501 (4)
C6A—H61A	0.9500	C6B—H61B	0.9500
C7A—C8A	1.523 (5)	C7B—C8B	1.524 (4)
C7A—H71A	0.9900	C7B—H71B	0.9900
C7A—H72A	0.9900	C7B—H72B	0.9900
C8A—C14A	1.524 (4)	C8B—C14B	1.525 (4)
C8A—C9A	1.542 (4)	C8B—C9B	1.532 (4)
C8A—H81A	1.0000	C8B—H81B	1.0000
C9A—C11A	1.538 (4)	C9B—C11B	1.535 (4)
C9A—C10A	1.552 (4)	C9B—C10B	1.559 (4)
C9A—H91A	1.0000	C9B—H91B	1.0000
C10A—C19A	1.542 (4)	C10B—C19B	1.544 (4)
C11A—C12A	1.536 (4)	C11B—C12B	1.540 (4)
C11A—H11A	0.9900	C11B—H11B	0.9900
C11A—H11C	0.9900	C11B—H11E	0.9900
C12A—C13A	1.528 (4)	C12B—C13B	1.531 (4)
C12A—H12A	0.9900	C12B—H12B	0.9900
C12A—H12C	0.9900	C12B—H12E	0.9900
C13A—C14A	1.541 (4)	C13B—C18B	1.535 (4)
C13A—C18A	1.541 (4)	C13B—C14B	1.543 (4)
C13A—C17A	1.551 (4)	C13B—C17B	1.556 (4)
C14A—C15A	1.522 (4)	C14B—C15B	1.523 (4)
C14A—H14A	1.0000	C14B—H14B	1.0000
C15A—C16A	1.546 (4)	C15B—C16B	1.550 (4)
C15A—H15A	0.9900	C15B—H15B	0.9900
C15A—H15C	0.9900	C15B—H15E	0.9900
C16A—C17A	1.557 (5)	C16B—C17B	1.548 (4)
C16A—H16A	0.9900	C16B—H16B	0.9900
C16A—H16C	0.9900	C16B—H16E	0.9900
C17A—C20A	1.527 (4)	C17B—C20B	1.531 (4)
C17A—H17A	1.0000	C17B—H17B	1.0000
C18A—H18A	0.9800	C18B—H18B	0.9800
C18A—H18C	0.9800	C18B—H18E	0.9800
C18A—H18D	0.9800	C18B—H18F	0.9800
C19A—H19A	0.9800	C19B—H19B	0.9800
C19A—H19C	0.9800	C19B—H19E	0.9800
C19A—H19D	0.9800	C19B—H19F	0.9800
C20A—C22A	1.517 (4)	C20B—C22B	1.523 (4)
C20A—C21A	1.533 (5)	C20B—C21B	1.535 (4)
C20A—H20A	1.0000	C20B—H20B	1.0000
C21A—H21A	0.9800	C21B—H21B	0.9800
C21A—H21C	0.9800	C21B—H21E	0.9800
C21A—H21D	0.9800	C21B—H21F	0.9800
C23A—H23A	0.9800	C23B—H23B	0.9800
C23A—H23C	0.9800	C23B—H23E	0.9800
C23A—H23D	0.9800	C23B—H23F	0.9800
C24A—H24A	0.9800	C24B—H24B	0.9800
C24A—H24C	0.9800	C24B—H24E	0.9800
C24A—H24D	0.9800	C24B—H24F	0.9800
			
N1A—O2A—C24A	109.5 (2)	N1B—O2B—C24B	109.7 (2)
C3A—O3A—H3A	107 (3)	C3B—O3B—H3B	107 (2)
C22A—N1A—O2A	117.5 (2)	C22B—N1B—O2B	116.2 (2)
C22A—N1A—C23A	123.6 (3)	C22B—N1B—C23B	121.2 (2)
O2A—N1A—C23A	113.3 (2)	O2B—N1B—C23B	112.2 (2)
C2A—C1A—C10A	114.9 (3)	C2B—C1B—C10B	114.4 (3)
C2A—C1A—H1A	108.5	C2B—C1B—H1B	108.7
C10A—C1A—H1A	108.5	C10B—C1B—H1B	108.7
C2A—C1A—H2A	108.5	C2B—C1B—H2B	108.7
C10A—C1A—H2A	108.5	C10B—C1B—H2B	108.7
H1A—C1A—H2A	107.5	H1B—C1B—H2B	107.6
C3A—C2A—C1A	110.0 (3)	C3B—C2B—C1B	110.3 (3)
C3A—C2A—H4A	109.7	C3B—C2B—H4B	109.6
C1A—C2A—H4A	109.7	C1B—C2B—H4B	109.6
C3A—C2A—H5A	109.7	C3B—C2B—H5B	109.6
C1A—C2A—H5A	109.7	C1B—C2B—H5B	109.6
H4A—C2A—H5A	108.2	H4B—C2B—H5B	108.1
O3A—C3A—C2A	108.2 (3)	O3B—C3B—C2B	112.1 (3)
O3A—C3A—C4A	111.5 (3)	O3B—C3B—C4B	107.1 (2)
C2A—C3A—C4A	110.5 (3)	C2B—C3B—C4B	110.7 (2)
O3A—C3A—H31A	108.9	O3B—C3B—H31B	109.0
C2A—C3A—H31A	108.9	C2B—C3B—H31B	109.0
C4A—C3A—H31A	108.9	C4B—C3B—H31B	109.0
C5A—C4A—C3A	113.1 (3)	C3B—C4B—C5B	112.5 (3)
C5A—C4A—H41A	109.0	C3B—C4B—H41B	109.1
C3A—C4A—H41A	109.0	C5B—C4B—H41B	109.1
C5A—C4A—H42A	109.0	C3B—C4B—H42B	109.1
C3A—C4A—H42A	109.0	C5B—C4B—H42B	109.1
H41A—C4A—H42A	107.8	H41B—C4B—H42B	107.8
C6A—C5A—C4A	120.8 (3)	C6B—C5B—C4B	119.8 (3)
C6A—C5A—C10A	123.3 (3)	C6B—C5B—C10B	123.4 (3)
C4A—C5A—C10A	115.8 (3)	C4B—C5B—C10B	116.8 (3)
C5A—C6A—C7A	124.9 (3)	C5B—C6B—C7B	124.9 (3)
C5A—C6A—H61A	117.5	C5B—C6B—H61B	117.6
C7A—C6A—H61A	117.5	C7B—C6B—H61B	117.6
C6A—C7A—C8A	112.8 (3)	C6B—C7B—C8B	113.5 (3)
C6A—C7A—H71A	109.0	C6B—C7B—H71B	108.9
C8A—C7A—H71A	109.0	C8B—C7B—H71B	108.9
C6A—C7A—H72A	109.0	C6B—C7B—H72B	108.9
C8A—C7A—H72A	109.0	C8B—C7B—H72B	108.9
H71A—C7A—H72A	107.8	H71B—C7B—H72B	107.7
C7A—C8A—C14A	111.2 (3)	C7B—C8B—C14B	110.3 (3)
C7A—C8A—C9A	109.8 (2)	C7B—C8B—C9B	110.5 (2)
C14A—C8A—C9A	110.1 (3)	C14B—C8B—C9B	109.3 (2)
C7A—C8A—H81A	108.6	C7B—C8B—H81B	108.9
C14A—C8A—H81A	108.6	C14B—C8B—H81B	108.9
C9A—C8A—H81A	108.6	C9B—C8B—H81B	108.9
C11A—C9A—C8A	112.2 (2)	C8B—C9B—C11B	111.4 (2)
C11A—C9A—C10A	113.3 (3)	C8B—C9B—C10B	113.3 (2)
C8A—C9A—C10A	112.4 (2)	C11B—C9B—C10B	113.1 (2)
C11A—C9A—H91A	106.1	C8B—C9B—H91B	106.1
C8A—C9A—H91A	106.1	C11B—C9B—H91B	106.1
C10A—C9A—H91A	106.1	C10B—C9B—H91B	106.1
C5A—C10A—C1A	108.6 (2)	C5B—C10B—C1B	108.9 (2)
C5A—C10A—C19A	108.0 (3)	C5B—C10B—C19B	108.6 (3)
C1A—C10A—C19A	109.3 (3)	C1B—C10B—C19B	109.4 (3)
C5A—C10A—C9A	110.1 (3)	C5B—C10B—C9B	109.8 (3)
C1A—C10A—C9A	109.1 (2)	C1B—C10B—C9B	108.9 (2)
C19A—C10A—C9A	111.7 (2)	C19B—C10B—C9B	111.3 (2)
C12A—C11A—C9A	114.9 (3)	C9B—C11B—C12B	113.9 (3)
C12A—C11A—H11A	108.5	C9B—C11B—H11B	108.8
C9A—C11A—H11A	108.5	C12B—C11B—H11B	108.8
C12A—C11A—H11C	108.5	C9B—C11B—H11E	108.8
C9A—C11A—H11C	108.5	C12B—C11B—H11E	108.8
H11A—C11A—H11C	107.5	H11B—C11B—H11E	107.7
C13A—C12A—C11A	112.0 (3)	C13B—C12B—C11B	112.2 (3)
C13A—C12A—H12A	109.2	C13B—C12B—H12B	109.2
C11A—C12A—H12A	109.2	C11B—C12B—H12B	109.2
C13A—C12A—H12C	109.2	C13B—C12B—H12E	109.2
C11A—C12A—H12C	109.2	C11B—C12B—H12E	109.2
H12A—C12A—H12C	107.9	H12B—C12B—H12E	107.9
C12A—C13A—C14A	106.7 (2)	C12B—C13B—C18B	110.6 (3)
C12A—C13A—C18A	110.5 (3)	C12B—C13B—C14B	106.6 (2)
C14A—C13A—C18A	112.0 (3)	C18B—C13B—C14B	112.6 (3)
C12A—C13A—C17A	116.5 (3)	C12B—C13B—C17B	117.4 (2)
C14A—C13A—C17A	100.5 (3)	C18B—C13B—C17B	110.2 (2)
C18A—C13A—C17A	110.1 (2)	C14B—C13B—C17B	98.9 (2)
C15A—C14A—C8A	118.6 (3)	C15B—C14B—C8B	118.5 (3)
C15A—C14A—C13A	104.6 (2)	C15B—C14B—C13B	105.0 (2)
C8A—C14A—C13A	114.6 (3)	C8B—C14B—C13B	115.3 (3)
C15A—C14A—H14A	106.1	C15B—C14B—H14B	105.6
C8A—C14A—H14A	106.1	C8B—C14B—H14B	105.6
C13A—C14A—H14A	106.1	C13B—C14B—H14B	105.6
C14A—C15A—C16A	103.7 (3)	C14B—C15B—C16B	103.9 (3)
C14A—C15A—H15A	111.0	C14B—C15B—H15B	111.0
C16A—C15A—H15A	111.0	C16B—C15B—H15B	111.0
C14A—C15A—H15C	111.0	C14B—C15B—H15E	111.0
C16A—C15A—H15C	111.0	C16B—C15B—H15E	111.0
H15A—C15A—H15C	109.0	H15B—C15B—H15E	109.0
C15A—C16A—C17A	107.0 (3)	C17B—C16B—C15B	106.3 (2)
C15A—C16A—H16A	110.3	C17B—C16B—H16B	110.5
C17A—C16A—H16A	110.3	C15B—C16B—H16B	110.5
C15A—C16A—H16C	110.3	C17B—C16B—H16E	110.5
C17A—C16A—H16C	110.3	C15B—C16B—H16E	110.5
H16A—C16A—H16C	108.6	H16B—C16B—H16E	108.7
C20A—C17A—C13A	117.8 (3)	C20B—C17B—C16B	112.4 (2)
C20A—C17A—C16A	111.6 (3)	C20B—C17B—C13B	119.5 (2)
C13A—C17A—C16A	104.0 (3)	C16B—C17B—C13B	103.6 (2)
C20A—C17A—H17A	107.7	C20B—C17B—H17B	106.9
C13A—C17A—H17A	107.7	C16B—C17B—H17B	106.9
C16A—C17A—H17A	107.7	C13B—C17B—H17B	106.9
C13A—C18A—H18A	109.5	C13B—C18B—H18B	109.5
C13A—C18A—H18C	109.5	C13B—C18B—H18E	109.5
H18A—C18A—H18C	109.5	H18B—C18B—H18E	109.5
C13A—C18A—H18D	109.5	C13B—C18B—H18F	109.5
H18A—C18A—H18D	109.5	H18B—C18B—H18F	109.5
H18C—C18A—H18D	109.5	H18E—C18B—H18F	109.5
C10A—C19A—H19A	109.5	C10B—C19B—H19B	109.5
C10A—C19A—H19C	109.5	C10B—C19B—H19E	109.5
H19A—C19A—H19C	109.5	H19B—C19B—H19E	109.5
C10A—C19A—H19D	109.5	C10B—C19B—H19F	109.5
H19A—C19A—H19D	109.5	H19B—C19B—H19F	109.5
H19C—C19A—H19D	109.5	H19E—C19B—H19F	109.5
C22A—C20A—C17A	109.9 (3)	C22B—C20B—C17B	109.1 (3)
C22A—C20A—C21A	106.3 (3)	C22B—C20B—C21B	107.5 (3)
C17A—C20A—C21A	114.6 (3)	C17B—C20B—C21B	113.6 (2)
C22A—C20A—H20A	108.6	C22B—C20B—H20B	108.8
C17A—C20A—H20A	108.6	C17B—C20B—H20B	108.8
C21A—C20A—H20A	108.6	C21B—C20B—H20B	108.8
C20A—C21A—H21A	109.5	C20B—C21B—H21B	109.5
C20A—C21A—H21C	109.5	C20B—C21B—H21E	109.5
H21A—C21A—H21C	109.5	H21B—C21B—H21E	109.5
C20A—C21A—H21D	109.5	C20B—C21B—H21F	109.5
H21A—C21A—H21D	109.5	H21B—C21B—H21F	109.5
H21C—C21A—H21D	109.5	H21E—C21B—H21F	109.5
O1A—C22A—N1A	119.4 (3)	O1B—C22B—N1B	119.1 (3)
O1A—C22A—C20A	122.3 (3)	O1B—C22B—C20B	122.5 (3)
N1A—C22A—C20A	118.3 (3)	N1B—C22B—C20B	118.4 (3)
N1A—C23A—H23A	109.5	N1B—C23B—H23B	109.5
N1A—C23A—H23C	109.5	N1B—C23B—H23E	109.5
H23A—C23A—H23C	109.5	H23B—C23B—H23E	109.5
N1A—C23A—H23D	109.5	N1B—C23B—H23F	109.5
H23A—C23A—H23D	109.5	H23B—C23B—H23F	109.5
H23C—C23A—H23D	109.5	H23E—C23B—H23F	109.5
O2A—C24A—H24A	109.5	O2B—C24B—H24B	109.5
O2A—C24A—H24C	109.5	O2B—C24B—H24E	109.5
H24A—C24A—H24C	109.5	H24B—C24B—H24E	109.5
O2A—C24A—H24D	109.5	O2B—C24B—H24F	109.5
H24A—C24A—H24D	109.5	H24B—C24B—H24F	109.5
H24C—C24A—H24D	109.5	H24E—C24B—H24F	109.5
			
C24A—O2A—N1A—C22A	112.3 (3)	C24B—O2B—N1B—C22B	122.9 (3)
C24A—O2A—N1A—C23A	−93.4 (3)	C24B—O2B—N1B—C23B	−91.7 (3)
C10A—C1A—C2A—C3A	−57.2 (4)	C10B—C1B—C2B—C3B	−58.0 (3)
C1A—C2A—C3A—O3A	178.6 (3)	C1B—C2B—C3B—O3B	177.7 (2)
C1A—C2A—C3A—C4A	56.3 (4)	C1B—C2B—C3B—C4B	58.2 (3)
O3A—C3A—C4A—C5A	−174.3 (3)	O3B—C3B—C4B—C5B	−176.0 (3)
C2A—C3A—C4A—C5A	−53.9 (4)	C2B—C3B—C4B—C5B	−53.5 (4)
C3A—C4A—C5A—C6A	−129.7 (3)	C3B—C4B—C5B—C6B	−131.5 (3)
C3A—C4A—C5A—C10A	50.5 (4)	C3B—C4B—C5B—C10B	48.4 (4)
C4A—C5A—C6A—C7A	−177.5 (3)	C4B—C5B—C6B—C7B	−179.6 (3)
C10A—C5A—C6A—C7A	2.3 (5)	C10B—C5B—C6B—C7B	0.5 (5)
C5A—C6A—C7A—C8A	12.6 (5)	C5B—C6B—C7B—C8B	11.6 (4)
C6A—C7A—C8A—C14A	−164.5 (3)	C6B—C7B—C8B—C14B	−160.5 (2)
C6A—C7A—C8A—C9A	−42.5 (3)	C6B—C7B—C8B—C9B	−39.6 (3)
C7A—C8A—C9A—C11A	−170.4 (3)	C7B—C8B—C9B—C11B	−172.9 (3)
C14A—C8A—C9A—C11A	−47.7 (3)	C14B—C8B—C9B—C11B	−51.4 (3)
C7A—C8A—C9A—C10A	60.5 (3)	C7B—C8B—C9B—C10B	58.2 (3)
C14A—C8A—C9A—C10A	−176.8 (2)	C14B—C8B—C9B—C10B	179.7 (2)
C6A—C5A—C10A—C1A	133.4 (3)	C6B—C5B—C10B—C1B	135.2 (3)
C4A—C5A—C10A—C1A	−46.8 (4)	C4B—C5B—C10B—C1B	−44.7 (3)
C6A—C5A—C10A—C19A	−108.2 (3)	C6B—C5B—C10B—C19B	−105.8 (3)
C4A—C5A—C10A—C19A	71.6 (3)	C4B—C5B—C10B—C19B	74.3 (3)
C6A—C5A—C10A—C9A	14.0 (4)	C6B—C5B—C10B—C9B	16.1 (4)
C4A—C5A—C10A—C9A	−166.2 (3)	C4B—C5B—C10B—C9B	−163.8 (2)
C2A—C1A—C10A—C5A	50.5 (4)	C2B—C1B—C10B—C5B	49.4 (3)
C2A—C1A—C10A—C19A	−67.1 (4)	C2B—C1B—C10B—C19B	−69.1 (3)
C2A—C1A—C10A—C9A	170.5 (3)	C2B—C1B—C10B—C9B	169.1 (3)
C11A—C9A—C10A—C5A	−173.5 (2)	C8B—C9B—C10B—C5B	−45.2 (3)
C8A—C9A—C10A—C5A	−45.0 (3)	C11B—C9B—C10B—C5B	−173.2 (2)
C11A—C9A—C10A—C1A	67.4 (3)	C8B—C9B—C10B—C1B	−164.3 (2)
C8A—C9A—C10A—C1A	−164.1 (3)	C11B—C9B—C10B—C1B	67.7 (3)
C11A—C9A—C10A—C19A	−53.5 (4)	C8B—C9B—C10B—C19B	75.1 (3)
C8A—C9A—C10A—C19A	75.0 (3)	C11B—C9B—C10B—C19B	−52.9 (3)
C8A—C9A—C11A—C12A	47.0 (4)	C8B—C9B—C11B—C12B	51.4 (3)
C10A—C9A—C11A—C12A	175.6 (3)	C10B—C9B—C11B—C12B	−179.6 (2)
C9A—C11A—C12A—C13A	−52.4 (4)	C9B—C11B—C12B—C13B	−54.1 (3)
C11A—C12A—C13A—C14A	56.0 (3)	C11B—C12B—C13B—C18B	−68.3 (3)
C11A—C12A—C13A—C18A	−66.0 (3)	C11B—C12B—C13B—C14B	54.4 (3)
C11A—C12A—C13A—C17A	167.3 (3)	C11B—C12B—C13B—C17B	164.0 (3)
C7A—C8A—C14A—C15A	−56.5 (4)	C7B—C8B—C14B—C15B	−54.4 (4)
C9A—C8A—C14A—C15A	−178.3 (3)	C9B—C8B—C14B—C15B	−176.1 (3)
C7A—C8A—C14A—C13A	179.2 (2)	C7B—C8B—C14B—C13B	179.9 (2)
C9A—C8A—C14A—C13A	57.4 (3)	C9B—C8B—C14B—C13B	58.3 (3)
C12A—C13A—C14A—C15A	167.5 (2)	C12B—C13B—C14B—C15B	168.6 (2)
C18A—C13A—C14A—C15A	−71.4 (3)	C18B—C13B—C14B—C15B	−69.9 (3)
C17A—C13A—C14A—C15A	45.5 (3)	C17B—C13B—C14B—C15B	46.4 (3)
C12A—C13A—C14A—C8A	−61.0 (3)	C12B—C13B—C14B—C8B	−59.0 (3)
C18A—C13A—C14A—C8A	60.1 (3)	C18B—C13B—C14B—C8B	62.4 (3)
C17A—C13A—C14A—C8A	177.0 (2)	C17B—C13B—C14B—C8B	178.8 (2)
C8A—C14A—C15A—C16A	−164.3 (3)	C8B—C14B—C15B—C16B	−162.5 (3)
C13A—C14A—C15A—C16A	−35.2 (3)	C13B—C14B—C15B—C16B	−32.0 (3)
C14A—C15A—C16A—C17A	11.0 (3)	C14B—C15B—C16B—C17B	4.4 (3)
C12A—C13A—C17A—C20A	83.7 (3)	C15B—C16B—C17B—C20B	154.5 (2)
C14A—C13A—C17A—C20A	−161.5 (3)	C15B—C16B—C17B—C13B	24.2 (3)
C18A—C13A—C17A—C20A	−43.2 (4)	C12B—C13B—C17B—C20B	77.7 (3)
C12A—C13A—C17A—C16A	−152.3 (3)	C18B—C13B—C17B—C20B	−50.2 (4)
C14A—C13A—C17A—C16A	−37.4 (3)	C14B—C13B—C17B—C20B	−168.3 (3)
C18A—C13A—C17A—C16A	80.9 (3)	C12B—C13B—C17B—C16B	−156.4 (3)
C15A—C16A—C17A—C20A	144.9 (2)	C18B—C13B—C17B—C16B	75.7 (3)
C15A—C16A—C17A—C13A	16.9 (3)	C14B—C13B—C17B—C16B	−42.5 (3)
C13A—C17A—C20A—C22A	178.5 (3)	C16B—C17B—C20B—C22B	58.9 (3)
C16A—C17A—C20A—C22A	58.3 (3)	C13B—C17B—C20B—C22B	−179.5 (3)
C13A—C17A—C20A—C21A	−62.0 (4)	C16B—C17B—C20B—C21B	178.9 (3)
C16A—C17A—C20A—C21A	177.9 (3)	C13B—C17B—C20B—C21B	−59.5 (4)
O2A—N1A—C22A—O1A	166.4 (3)	O2B—N1B—C22B—O1B	162.6 (3)
C23A—N1A—C22A—O1A	14.9 (5)	C23B—N1B—C22B—O1B	20.5 (5)
O2A—N1A—C22A—C20A	−14.0 (4)	O2B—N1B—C22B—C20B	−18.9 (4)
C23A—N1A—C22A—C20A	−165.5 (3)	C23B—N1B—C22B—C20B	−161.0 (3)
C17A—C20A—C22A—O1A	37.4 (4)	C17B—C20B—C22B—O1B	27.8 (4)
C21A—C20A—C22A—O1A	−87.2 (4)	C21B—C20B—C22B—O1B	−95.8 (4)
C17A—C20A—C22A—N1A	−142.2 (3)	C17B—C20B—C22B—N1B	−150.7 (3)
C21A—C20A—C22A—N1A	93.2 (3)	C21B—C20B—C22B—N1B	85.8 (3)

### Hydrogen-bond geometry (Å, º)

**Table:**

*D*—H···*A*	*D*—H	H···*A*	*D*···*A*	*D*—H···*A*
O3*A*—H3*A*···O3*B*^i^	0.86 (4)	1.93 (4)	2.782 (4)	180 (5)
O3*B*—H3*B*···O1*A*^ii^	0.83 (4)	1.95 (4)	2.768 (3)	169 (4)

Symmetry codes: (i) −*x*+3/2, −*y*+1, *z*−1/2; (ii) −*x*+1, *y*−1/2, −*z*+3/2.
